# Revealing RNA virus diversity and evolution in unicellular algae transcriptomes

**DOI:** 10.1093/ve/veab070

**Published:** 2021-08-14

**Authors:** Justine Charon, Shauna Murray, Edward C Holmes

**Affiliations:** Marie Bashir Institute for Infectious Diseases and Biosecurity, School of Life and Environmental Sciences and School of Medical Sciences, The University of Sydney, Sydney, NSW 2006, Australia; School of Life Sciences, University of Technology Sydney, Sydney, NSW 2007, Australia; Marie Bashir Institute for Infectious Diseases and Biosecurity, School of Life and Environmental Sciences and School of Medical Sciences, The University of Sydney, Sydney, NSW 2006, Australia

**Keywords:** RNA virus, protist RNA viruses, algae viruses, virus evolution, virus metatranscriptomics

## Abstract

Remarkably little is known about the diversity and evolution of RNA viruses in unicellular eukaryotes. We screened a total of 570 transcriptomes from the Marine Microbial Eukaryote Transcriptome Sequencing Project that encompasses a wide diversity of microbial eukaryotes, including most major photosynthetic lineages (i.e. the microalgae). From this, we identified thirty new and divergent RNA virus species, occupying a range of phylogenetic positions within the overall diversity of RNA viruses. Approximately one-third of the newly described viruses comprised single-stranded positive-sense RNA viruses from the order *Lenarviricota* associated with fungi, plants, and protists, while another third were related to the order *Ghabrivirales*, including members of the protist and fungi-associated *Totiviridae*. Other viral species showed sequence similarity to positive-sense RNA viruses from the algae-associated *Marnaviridae*, the double-stranded RNA (ds-RNA) *Partitiviridae*, as well as tentative evidence for one negative-sense RNA virus related to the *Qinviridae*. Importantly, we were able to identify divergent RNA viruses from distant host taxa, revealing the ancestry of these viral families and greatly extending our knowledge of the RNA viromes of microalgal cultures. Both the limited number of viruses detected per sample and the low sequence identity to known RNA viruses imply that additional microalgal viruses exist that could not be detected at the current sequencing depth or were too divergent to be identified using sequence similarity. Together, these results highlight the need for further investigation of algal-associated RNA viruses as well as the development of new tools to identify RNA viruses that exhibit very high levels of sequence divergence.

## 1. Introduction

Metagenomic studies of marine environments have revealed an enormous abundance and diversity of both DNA and RNA viruses (up to 10^8^ viruses/ml) (Wigington et al. 2016) and shown that they play a key role in biogeochemical processes (Suttle 2007). Such ubiquity highlights the value of obtaining a comprehensive picture of global virus diversity, including in host taxa that have been poorly sampled to date (Dolja and Koonin 2018). Viruses of protists are an important exemplar of this untapped diversity.

**Figure 1.**
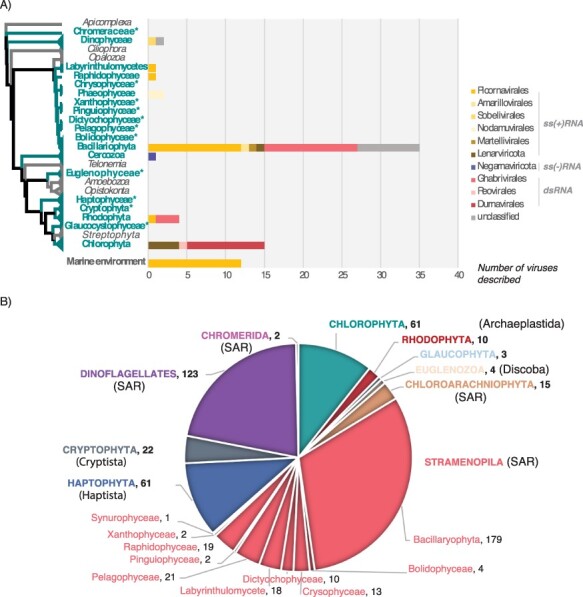
Currently reported RNA virus diversity in microalgae and the taxa studied here. (A) Left, eukaryote cladogram from Burki et al. (2020). The microalgae-containing eukaryotic lineages investigated here are highlighted in bold green. *Microalgae lineages for which no RNA viruses have been reported to date. Right, number of total RNA viruses formally or likely associated with microalgae reported at NCBI (https://www.ncbi.nlm.nih.gov/labs/virus/vssi/), VirusHostdb (https://www.genome.jp/virushostdb/) and the literature. Viruses are coloured based on their taxonomy and genome composition. (B) Representative taxa from major algal lineages used in this study and the total number of transcriptomes analysed for each lineage. Newly established eukaryotic supergroups (Burki et al. 2020) are indicated by brackets.

Protists, defined as eukaryotic organisms that are not animal, plant, or fungi, comprise most of the diversity of eukaryotes and are distributed among each of the newly established eukaryotic supergroups (Burki et al. 2020). Some protists, especially microalgae, play a critical role in ecosystems as primary producers as well as being involved in nutrient cycling. Next-generation sequencing (NGS) has revealed that the diversity of protists is far greater than previously thought, with species numbers likely exceeding 1 million, although only a tiny fraction have been described to date (Pawlowski et al. 2012). Protists have already proven to be an important source of virus diversity, with the giant *Mimiviridae* from the Amoebozoa a notable case in point (Raoult and Forterre 2008). Despite this, protist viruses remain largely overlooked, especially those associated with unicellular microalgae. This is particularly striking in the case of RNA viruses: although RNA viruses were first described in unicellular algae in 2003 (Tai et al. 2003), they still comprise only 73 species sampled from a very small number of algal lineages (Short et al. 2020) (Fig. 1).

There have been several metagenomic studies of viruses in aquatic microbial eukaryotes (Brum et al. 2015; Gregory et al. 2019), identifying many thousands of virus sequences, with at least half predicted to have RNA genomes (Steward et al. 2013; Wolf et al. 2020). Similarly, metagenomics is proving a valuable way to mine viral diversity in uncultivable organisms (Simmonds et al. 2017). However, because these studies have been conducted with environmental samples, they cannot identify the specific host taxon with certainty. This illustrates the inference gap between broad metagenomic surveys that identify huge numbers of new viral sequences, creating a large but unassigned depiction of the virosphere, and studies based on virus isolation and detailed particle characterization (including cell culture) that are conducted on a very limited number of viruses and create a highly accurate, but very narrow, vision of the virosphere (Nissimov et al. 2020). The NGS-based investigation of RNA virus diversity from individual host species therefore serves as a good compromise to fill the gap between large-scale virus detection through metagenomics and the detailed assignment of hosts through virus isolation.

To better understand the diversity of RNA viruses associated with microalgae, we performed viral metatranscriptomic analyses of data obtained from the Marine Microbial Eukaryote Transcriptome Sequencing Project (MMETSP) (Keeling et al. 2014). With 210 unique genera covering most unicellular algal-comprising lineages, the MMETSP constitutes the largest collection of transcriptome data collected from microbial eukaryote cultures, including axenic ones, and depicts a large component of eukaryotic diversity (Keeling et al. 2014) (Fig. 1). We used both sequence- and structural-based approaches to screen 570 transcriptomes from nineteen major microalgae-containing lineages for the most conserved ‘hallmark’ protein of RNA viruses—the RNA-dependent RNA polymerase (RdRp). To the best of our knowledge, this is the broadest exploration of RNA viruses conducted at the level of single host species in microbial eukaryotes and the first attempt to identify RNA viruses in most of the microalgal lineages investigated (Fig. 1).

## 2. Methods

### 2.1 MMETSP contig retrieval

In total, 570 MMETSP accessions, corresponding to the microalgal-containing lineages, were included in this study. Contig data sets corresponding to each accession were retrieved from a Trinity re-assembly performed on the RNA-Seq data sets from MMETSP and available at http://10.5281/zenodo.740440 (Johnson, Alexander, and Brown 2019). A description of all the transcriptome accessions and samples analysed here is available in Table S1.

### 2.2 ORF annotation

To optimize our computational analysis of the 570 contig data sets, we focused on those predicted to encode Open reading frames (ORFs) with a minimum length of 200 amino acids (assuming that shorter contigs would not result in robust phylogenetic analyses). Accordingly, ORFs >200 amino acids in length were predicted using the GetORF tool from the EMBOSS package (v6.6.0). ORFs were predicted using the standard genetic code as alternative genetic codes are not used in the microalgae analysed here (Swart et al. 2016). The option -find 0 (translation of regions between STOP codons) was used to enable the detection of partial genomes, in which START codons could be missing due to partial virus genome recovery.

### 2.3 RNA virus sequence detection using sequence similarity

All predicted ORFs were compared to the entire non-redundant (nr) protein database (release April 2020) using DIAMOND BLASTp (v0.9.32) (Buchfink, Xie, and Huson 2015), with the following options: --max-target-seqs 1 (top hit with best score retained) and an e-value cut-off of 1e-03. Additional sequence comparisons with identical BLASTp parameters were performed using either the newly detected RdRp sequences or the RdRps from a previous large-scale analysis (Wolf et al. 2020) (available at ftp://ftp.ncbi.nih.gov/pub/wolf/_suppl/yangshan/rdrp.ya.fa).

To limit false-negative detection due to a bias in ORF prediction (in particular, partial genomes may not be detected due to their short length), all contig nucleotide sequences were submitted to a RdRp protein database using DIAMOND BLASTx (v0.9.32, more sensitive option and 1e-03 e-value cut-off) (Buchfink, Xie, and Huson 2015) to identify any additional RNA viruses. Top hits were retained and re-submitted against the entire nr protein database to remove false-positive hits (queries with a greater match to non-viral hits). All sequences retained from both the BLASTp and RdRp BLASTx analyses were manually checked to remove non-RNA virus sequences based on their taxonomy (predicted using the TaxonKit tool from NCBI; https://github.com/shenwei356/taxonkit).

All RNA virus-like sequences detected were functionally annotated using InterProscan (v5.39-77.0, default parameters), and non-RdRp sequences were filtered out. One sequence, sharing homology with the QDH87844.1 hypothetical protein H3RhizoLitter144407_000001, partial [Mitovirus sp.], was observed in eighty-six of the 570 data sets, including multiple species from multiple sampling locations. Considering its prevalence and 100 per cent identity between samples, we assumed this originates from environmental or sequencing-associated contamination. A small number of RNA virus-like sequences with homology to bovine viral diarrhoea viruses 1 and 2 were similarly considered biological product contaminants (Giangaspero 2013).

### 2.4 RNA virus sequence detection using protein profiles and three-dimensional structures

To detect more divergent viral RdRps, we compared all the ‘orphan’ ORFs (i.e. ORFs without any BLASTp hits at the 1e-03 e-value cut-off) against the viral RdRp-related profiles from the PFAM (El-Gebali et al. 2019) and PROSITE databases (Table S2) using the HMMer3 program (Eddy 2011) (v3.3, default parameters, e-value <1e-05). An additional attempt to annotate orphan translated-ORFs was performed on the remaining sequences using the InterProscan software package from EMBL-EBI (v5.39-77.0, default parameters) (https://github.com/ebi-pf-team/interproscan).

The RdRp-like candidates identified in the HMMer3 and InterProscan analyses were submitted to the Protein Homology/analogY Recognition Engine v 2.0 (Phyre2) web portal (Kelley et al. 2015) to confirm the presence of a RdRp signature (Table S3). Non-viral proteins (i.e. non-viral Phyre2 hit >90 per cent confidence) were discarded, as were sequences with low HMM (e-value >1e-03) and Phyre2 scores (confidence level >90 per cent). Sequences that matched either the HMM RdRp (>1e-05) and/or Phyre2 RdRp (>90 per cent confidence) were retained for further characterization. In total, eighty RdRp-like candidates were quality-assessed by coverage analysis and manually checked for the presence of the standard A, B, and C catalytic viral RdRp sequence motifs (Venkataraman, Prasad, and Selvarajan 2018) using Geneious (v11.1.4) (Kearse et al. 2012). Only those displaying related RdRp-like motifs were retained as potential RdRp protein candidates (Table S3).

### 2.5 Contig manual extension and genome annotation

Full-length nucleotide sequences encoding the protein retained from the sequence-based and structure-based detection approaches were retrieved and used as references for mapping Sequence Read Archive (SRA) reads corresponding to each sample (BioProject PRJNA231566) using the SRA extension package of Bowtie2 (v2.3.5.1-sra) (Langmead and Salzberg 2012). Read coverages of each contig were checked using Geneious (v11.1.4), and when needed, extremities were manually extended and contigs re-submitted to read mapping.

The relative abundance of each putative viral sequence was reported as the number of reads per million (i.e. the number of reads mapping to the contig divided by the total number of reads of the corresponding SRA library multiplied by 1 million). Poorly represented viral sequences were considered as potential cross-library contaminants derived from index-hopping and discarded when they accounted for less than 0.1 per cent of the highest abundance of the same sequence in another library (Pettersson et al. 2020).

Genomic organizations were inferred using Geneious (v11.1.4). ORFs were predicted using the standard genetic code or, when suitable, using alternative mitochondrial or plastid-associated genetic codes. Tentative virus names were taken from Greek mythology.

### 2.6 Host *rbcL* gene abundance estimation

To estimate levels of virus abundance in comparison to those from their putative hosts, the abundance of the host Ribulose bisphosphate carboxylase large chain (*rbcL*) gene was assessed using the Bowtie2 SRA package (v2.3.5.1-sra) and mapped to SRA reads from the *rbcL* gene of each corresponding species (whenever available) (Langmead and Salzberg 2012). The SRA and *rbcL* gene accessions used are reported in Table S4.

### 2.7 Secondary host profiling

All MMETSP cultures were subjected to Small subunit ribosomal RNA (SSU rRNA) sequencing to ensure they were mono-strain and not contaminated with additional microbial eukaryotes. Nevertheless, the presence of other microbial contaminants was possible. Assuming that most of the Archaea and Bacteria potential contaminants do not have an available genome sequence, the detection of contaminants was performed by analysing the closest homologs of each contig using both BLASTn (BLAST+ package, v2.9.0) and BLASTp (DIAMOND, v2.0.4) against the nt and nr databases, respectively. Contigs were grouped at the kingdom level based on the taxonomic affiliation of their closest homologs in the databases, with the abundance of each kingdom defined as the sum of each contig abundance value (transcripts per million) (Johnson, Alexander, and Brown 2019).

### 2.8 Phylogenetic analysis

**Figure 5.**
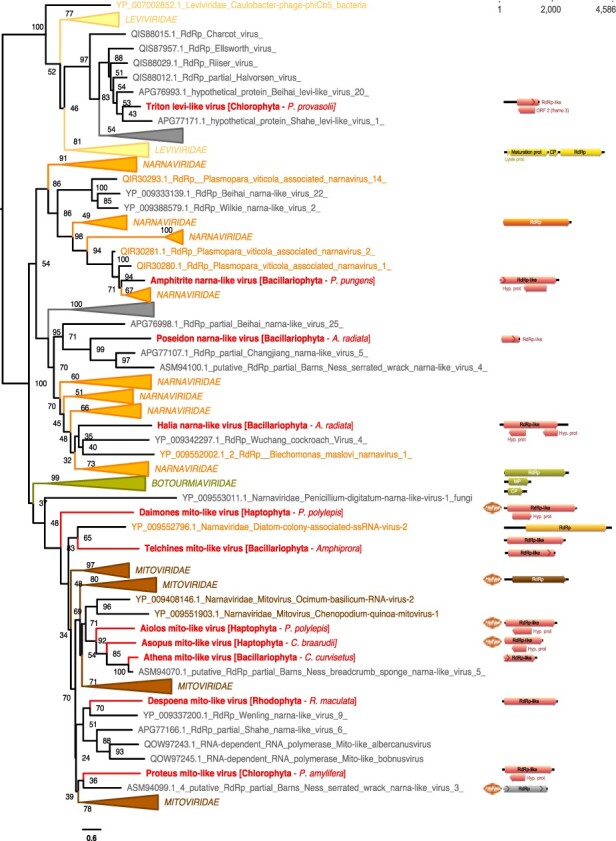
Phylogenetic position of the newly described RNA virus sequences in the phylum *Lenarviricota*. Left: Maximum likelihood (ML) phylogeny of the *Lenaviricota* RdRp (LG + F + R8 amino acid substitution model). Newly described viruses are shown in red. Algal host taxa are specified in brackets. Branch labels = bootstrap support (%). The tree is mid-point rooted for clarity only. Right: genomic organization of the newly described viruses (red), closest homologs, and *Lenarviricota* RefSeq representatives: Cassava virus C (NC_013111; *Botourmiaviridae*), Saccharomyces 23S RNA (NC_004050; *Narnaviridae*), Acinetobacter phage AP205 (NC_002700; *Leviviridae*), and Chenopodium quinoa mitovirus 1 (NC_040543; *Mitoviridae*). ORFs translated with the mitochondrial genetic code are marked a mitochondria icon. For clarity, some lineages were collapsed (a non-collapsed version of the tree is available as Supplementary Information).

**Figure 8.**
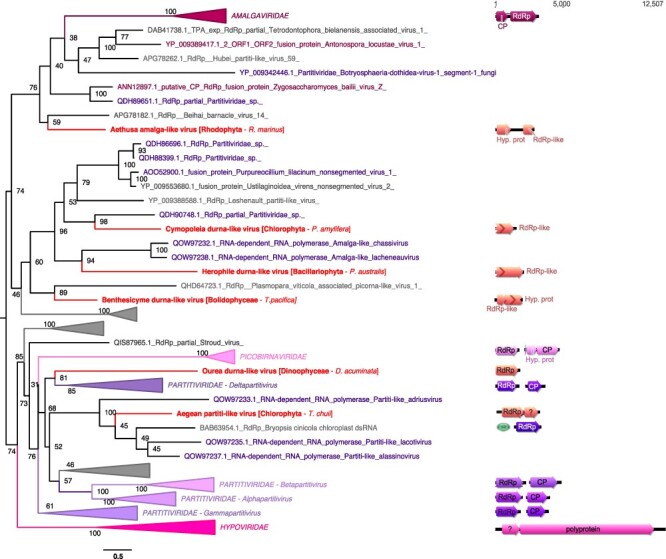
Phylogenetic positions of the newly described RNA viruses among the *Durnavirales*. Left, Maximum likelihood (ML) phylogeny of the *Durnavirales* RdRp (LG + F + R8 amino acid substitution model). Newly described viruses are indicated in red. Algae host taxon and species are specified in brackets. Branch labels = bootstrap support (%). The trees are mid-point rooted for clarity only. Right, genomic organization of newly discovered viruses (red), closest homologs, and the following Partiti-picobirna super-clade representatives: Zygosaccharomyces bailii virus Z (NC_003874; *Amalgaviridae*), Cryphonectria hypovirus 2 (NC_003534; *Hypoviridae*), Chicken picornavirus (NC_003534/NC_040438; *Picobirnaviridae*), Fig cryptic virus (NC_015494/NC_015495; *Deltapartitivirus*), Discula destructiva virus 1 (NC_002797/NC_002800; *Gammapartitivirus*), Ceratocystis resinifera virus 1 (NC_010755/NC_010754; *Betapartitivirus*), and White clover cryptic virus 1 (NC_006275/NC_006276; *Alphapartitivirus*). ORFs translated with the plastid genetic code are labelled with a green plastid. For clarity, some lineages were collapsed (a non-collapsed version of the tree is available as Supplementary Information).

For each virus phylum and order, the RefSeq and most closely related RdRp sequences were retrieved from GenBank and aligned with newly identified RdRp sequences using the L-INS-I algorithm in the MAFFT program (v7.402) (Katoh and Standley 2013). The resulting sequence alignments were trimmed using TrimAl to remove ambiguously aligned regions with different levels of stringency, optimized for each alignment (v1.4.1, ‘automated1’ mode). Maximum likelihood phylogenies based on amino acid alignments were inferred using IQ-TREE (v2.0-rc1) (Nguyen et al. 2015), with ModelFinder used to find the best-fit substitution model in each case (see figures 5 and 8 legends) (Kalyaanamoorthy et al. 2017) and both the SH-like approximate likelihood ratio test and ultrafast nonparametric bootstrap (1,000 replicates) used to assign support to individual nodes (Minh, Nguyen, and Von Haeseler 2013). All phylogenies were visualized and mid-point rooted (for clarity only) using Figtree (v1.4.4; http://tree.bio.ed.ac.uk/software/figtree/).

### 2.9 Detection of endogenous viral elements

To determine whether any of the newly detected viral sequences were likely endogenous viral elements (EVEs) rather than exogenous viruses, the nucleotide sequences of viral candidates were used as queries for BLASTn searches against the corresponding host genome sequence when available.

## 3. Results

### 3.1 Overall virus diversity

**Figure 2.**
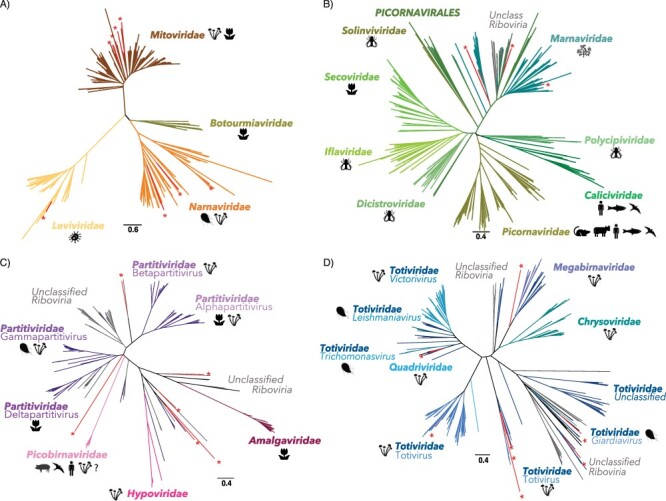
Newly described RNA virus sequences within the diversity of RNA viruses using RdRp phylogenies. Newly described sequences are indicated in red with ‘*’ symbols. Phylogenies of (A) the phylum Lenarnaviricota (ssRNA+), (B) the order Picornavirales (ssRNA+), (C) the order Durnavirales (dsRNA), and (D) the order Ghabrivirales (dsRNA). For each viral family, the host range was retrieved from VirusHostdb and the ICTV report.

Our analysis of the 570 MMETSP transcriptomes from 247 total microalgal species spread, over ten major groups of algae (Table 1) identified thirty new RNA viral species. These largely represented the single-stranded positive-sense RNA (ssRNA+) virus phylum *Lenarviricota* and the order *Picornavirales* (Fig. 2A, B), as well as the dsRNA virus orders *Durnavirales* and *Ghabrivirales* (Fig. 2C, D). A single negative-sense RNA virus was also identified in *Pseudo-nitzchia heimii* that fell within the *Qinviridae* (order *Muvirales*). Notably, all the RdRps identified in the BLAST analysis exhibited very high levels of sequence divergence, with median pairwise identity values of only ∼35 per cent to the closest known virus homolog (Table 1). In addition, with the exceptions of Pelias marna-like virus and Neleus marna-like virus, the newly described viral sequences were at relatively low abundance all (Table 1). This may reflect the lack of an rRNA depletion step used in the MMETSP library preparation, such that any RNA viruses would necessarily only represent a small proportion of reads. To shed more light on this issue, we compared levels of virus abundance with the expression levels of a host gene, that encoding the large subunit of the ribulose-1,5-bisphosphate carboxylase/oxygenase (*rbcL*) (Fig. S1, Table S4), commonly used as a diversity marker in algae (John, Patterson, and Paul 2007). Overall, the number of reads mapping to putative RNA viruses are in the same order of magnitude or higher than those reported for the host *rbcL* gene (Fig. S1), compatible with their designation as replicating viruses.

**Table 1.** List of new RNA viruses discovered in this study. Read abundances are indicated as the number of reads per million. Likely hosts correspond to eukaryotic lineages detected using BLASTn/BLASTp analyses and phylogenies.

Virus name	MMETSP sample (phylum/class)	Genome status	Reads/million	BLASTp best hits (GenBank acc./organism)	%ID	e-value	Likely host(s) (BLAST)	Likely host(s) (phylogenies)	Proposed host
Amphitrite narna-like virus	MMETSP1061 *P. pungens* (Bacillariophyta)	Full-length	48	QIR30281.1 RdRp [Plasmopara viticola associated narnavirus 2]	41	5e-144	Bacillariophyta	Fungi/protist	Bacillariophyta
Poseidon narna-like virus	MMETSP0418 *A. radiata* (Bacillariophyta)	Partial	8	QDH89392.1 RdRp, partial [Mitovirus sp.]	34	4e-17	Bacillariophyta	Marine arthropod	Bacillariophyta
Halia narna-like virus	MMETSP0418 *A. radiata* (Bacillariophyta)	Full-length	108	QBC65281.1 RdRp, partial [Rhizopus microsporus 23S narnavirus]	32	4e-17	Bacillariophyta	Protist	Bacillariophyta
Triton levi-like virus	MMETSP1471 *P. provasolii* (Chlorophyta)	Partial	64	APG76993.1 hypothetical protein [Beihai levi-like virus 20]	46	3e-65	Chlorophyta; Bacteria	Bacteria	Bacteria
Aiolos mito-like virus	MMETSP0286 *P. polylepis* (Haptophyta)	Full-length	54	YP_009272901.1 RdRp [Fusarium poae mitovirus 4]	35	3e-38	Haptophyta	Sea sponge	Haptophyta
Asopus mito-like virus	MMETSP0164 *C. braarudii* (Haptophyta)	Partial	12	QDM55307.1 RdRp [Geopora sumneriana mitovirus 1]	34	2e-35	Haptophyta	Sea sponge	Haptophyta
Athena mito-like virus	MMETSP0719 *C. curvisetus* (Bacillariophyta)	Partial	54	ASM94070.1 putative RdRp, partial [Barns Ness breadcrumb sponge narna-like virus 5]	65	6e-72	Bacillariophyta; Bacteria	Sea sponge	Bacillariophyta
Daimones mito-like virus	MMETSP0286 *P. polylepis* (Haptophyta)	Full-length	104	YP_009552787.1 RNA-directed RNA polymerase [Rhizophagus sp. RF1 mitovirus]	26	4e-16	Haptophyta	Freshwater arthropods	Haptophyta
Despoena mito-like virus	MMETSP0167 *R. maculata* (Rhodophyta)	Full-length	115	ALM62241.1 RdRp [Soybean leaf-associated mitovirus 1]	34	6e-32	Rhodophyta; Bacteria	Freshwater arthropods	Rhodophyta
Proteus mito-like virus	MMETSP1081 *P. amylifera* (Chlorophyta)	Full-length	388	ALM62242.1 RdRp [Soybean leaf-associated mitovirus 2]	32	7e-46	Chlorophyta	Fungi/protist	Chlorophyta
Telchines mito-like virus	MMETSP0725 *Amphiprora* (Bacillariophyta)	Partial	15	QDA33961.1 RdRp [Mitovirus 1 BEG47]	25	5e-21	Bacillariophyta	Algae	Bacillariophyta
	MMETSP0724 *Amphiprora* (Bacillariophyta)	Partial	26						
Susy yue-like virus	MMETSP1423 *P. heimii* (Bacillariophyta)	Partial	5	QDH86724.1 RdRp, partial [Qinviridae sp.]	42	1e-21	Bacillariophyta	Soil samples/marine arthropod	Bacillariophyta
Aethusa amalga-like virus	MMETSP0011 *R. marinus* (Rhodophyta)	Partial	83	ANN12897.1 putative CP/RdRp [Zygosaccharomyces bailii virus Z]	43	2e-12	Rhodophyta; Bacteria	Marine arthropod	Rhodophyta
Benthesicyme durna-like virus	MMETSP1319 *T. pacifica* (Bolidophyceae)	Partial	404	QDH90748.1 RdRp, partial [Partitiviridae sp.]	29	1e-17	Bolidophyceae	Protist	Bolidophyceae
Herophile durna-like virus	MMETSP0140 *P. australis* (Bacillariophyta)	Partial	10	QOW97238.1 RdRp [Amalga-like lacheneauvirus]	27	2e-19	Bacillariophyta	Chlorophyta	Bacillariophyta
Cymopoleia durna-like virus	MMETSP1081 *P. amylifera* (Chlorophyta)	Partial	10	YP_009551448.1 RdRp [Diatom colony-associated dsRNA virus 2]	31	2e-34	Chlorophyta	Fungi	Chlorophyta
Ourea durna-like virus	MMETSP0797 *D. acuminata* (Dinophyceae)	Partial	4	ARO72610.1 RdRp [Spinach deltapartitivirus 1]	27	4e-11	Dinophyceae; Bacteria	Land plant	Dinophyceae
Aegean partiti-like virus	MMETSP0491 *T. chuii* (Chlorophyta)	Full-length	3,296	QOW97235.1 RdRp [Partiti-like lacotivirus]	29	6e-62	Chlorophyta	Chlorophyta	Chlorophyta
Pelias marna-like virus	MMETSP1377 *Symbiodinium sp.* (Dinophyceae)	Full-length	60,553	YP_009337401.1 hypothetical protein 2 [Wenzhou picorna-like virus 4]	26	8e-98	Dinophyceae	Algae	Xanthophyceae
Neleus marna-like virus, 1	MMETSP0946 *V. litorea* (Xanthophyceae)	Full-length	806,763	YP_009336927.1 hypothetical protein 1 [Shahe picorna-like virus 3]	33	3e-180	Vaucheriaceae	Algae	Xanthophyceae
Neleus marna-like virus, 2	MMETSP0945 *V. litorea* (Xanthophyceae)	Full-length	711,119	YP_009336927.1 hypothetical protein 1 [Shahe picorna-like virus 3]	33	4e-180	Vaucheriaceae	Algae	Xanthophyceae
Tyro marna-like virus	MMETSP0905 *T. antarctica* (Bacillariophyta)	Partial	126	YP_001429582.1 hypothetical protein JP-A_gp2 [Marine RNA virus JP-A]	75	3e-272	Bacillariophyta; Bacteria	Algae	Bacillariophyta
	MMETSP0903 *T. antarctica* (Bacillariophyta)	Partial	2,034						
	MMETSP0902 *T. antarctica* (Bacillariophyta)	Partial	237						
Aloadae toti-like virus, 1	MMETSP1388 *Isochrysis* (Haptophyta)	Partial	39	QIJ70132.1 RdRp [Keenan toti-like virus]	33	2e-109	Haptophyta	Fungi /invertebrates	Haptophyta
Aloadae toti-like virus, 2	MMETSP1090 *Isochrysis* (Haptophyta)	Partial	11	QIJ70132.1 RdRp [Keenan toti-like virus]	33	2e-109	Haptophyta	Fungi /invertebrates	Haptophyta
Antaeus toti-like virus, 1	MMETSP0154 *T. antarctica* (Bacillariophyta)	Full-length	27	QGY72637.1 putative CP [Plasmopara viticola associated totivirus-like 2]	22	1e-10	Bacillariophyta	Protist	Bacillariophyta
Antaeus toti-like virus, 2	MMETSP0152 *T. antarctica* (Bacillariophyta)	Full-length	7	BBJ21451.1 CP-RdRp fusion protein [Pythium splendens RNA virus 1]	40	5e-53	Bacillariophyta	Protist	Bacillariophyta
Charybdis toti-like virus	MMETSP0853 *P. fraudulenta* (Bacillariophyta)	Partial	38	YP_003288763.1 RdRp [Rosellinia necatrix megabirnavirus 1/W779]	30	4e-24	Bacillariophyta; Bacteria	Fungi	Bacillariophyta
	MMETSP0851 *P. fraudulenta* (Bacillariophyta)	Partial	44						
	MMETSP0850 *P. fraudulenta* (Bacillariophyta)	Partial	41						
	MMETSP0852 *P. fraudulenta* (Bacillariophyta)	Partial	14						
Chrysaor toti-like virus	MMETSP0418 *A. radiata* (Bacillariophyta)	Partial	40	YP_009551502.1 RdRp [Diatom colony-associated dsRNA virus 17 genome type B]	27	9e-95	Bacillariophyta; Bacteria	Soil	Bacillariophyta
Laestrygon toti-like virus	MMETSP1451 *V. brassicaformis* (Chromeraceae)	Partial	29	YP_009551504.1 RdRp [Diatom colony-associated dsRNA virus 17 genome type A]	34	4e-112	Chromeraceae	Soil	Chromeraceae
Arion toti-like virus	MMETSP0796 *P. bahamense* (Dinophyceae)	Partial	31	QGA70930.1 RdRp [Ahus virus]	25	3e-18	Dinophyceae; Bacteria	Protist/ marine host	Dinophyceae
Otus toti-like virus	MMETSP0011 *R. marinus* (Rhodophyta)	Full-length	31	AMB17469.1 RdRp, partial [Delisea pulchra totivirus IndA]	51	3e-120	Rhodophyta; Bacteria	Fungi	Rhodophyta
Polyphemus toti-like virus	MMETSP0418 *A. radiata* (Bacillariophyta)	Partial	10	YP_009552789.1 RdRp [Diatom colony-associated dsRNA virus 5]	59	3e-79	Bacillariophyta; Bacteria	Algae/ protist	Bacillariophyta
Ephialtes toti-like virus	MMETSP0418 *A. radiata* (Bacillariophyta)	Partial	13	YP_009552789.1 RdRp [diatom colony-associated dsRNA virus 5]	63	1e-200	Bacillariophyta; Bacteria	Algae/ protist	Bacillariophyta

### 3.2 Additional cellular organisms in the transcriptome data

**Figure 3.**
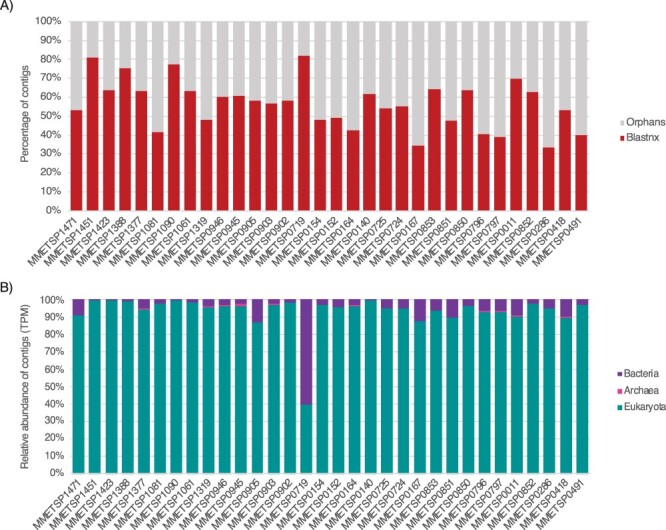
Taxonomic assignment of contigs in RNA virus-positive MMETSP libraries. (A) Ratio of contigs with hits to the nt and nr databases (red) versus orphans contigs (grey). (B) Relative abundance of cellular organism-like contigs based on the taxonomic assignment of their closest homologs in the nr and nt databases at the kingdom level. Contig abundances are calculated as transcripts per million (TPM).

We used mono-strain cultures of microbial eukaryotes to investigate the relationship between RNA viruses and their hosts. While the lack of additional eukaryotic organisms (fungi, other protists) was supposedly ensured under the MMETSP project guidelines with 18S rRNA sequencing of each culture (Keeling et al. 2014), some caveats remain for non-axenic cultures (Table S5). Indeed, some cultures likely contain contaminating Bacteria or Archaea, sometimes as intracellular parasites or as obligate mutualists in the culture media (Keeling et al. 2014). To assess this, contigs from libraries positive for RNA viruses were submitted to BLASTn and BLASTx. The ratio of assigned contigs and their kingdom assignments are summarized Fig. 3 and used to infer the likely host organisms (Table 1).

Approximately half of the total contigs identified here could not be assigned using BLAST approaches (Fig. 3A), with prokaryotic organisms on average representing less than 10 per cent of assigned contigs (Fig. 3B). However, the MMETSP0719 containing *Chaetoceros curvisetus* (Bacillariophyta) was enriched with co-infecting bacteria, largely due to the presence of the marine alphaproteobacteria *Jannaschia*. This is to be expected as some algal species require the presence of particular bacterial species to obtain essential nutrients (Bolch, Subramanian, and Green 2011).

### 3.3 Distribution and prevalence of RNA viruses in MMETSP cultured strains

**Figure 4.**
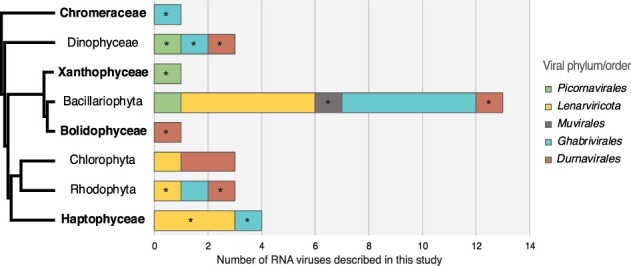
Distribution of RNA virus groups identified in algae. Only algal lineages containing RNA virus RdRps are shown. Left, cladogram of the algal host lineages positive for RNA viruses (retrieved from Burki et al. 2020). Taxa for which no RNA viruses have previously been reported are indicated in bold. Right, total counts of newly described RNA viral sequences in each algal taxon (including viruses observed in several samples from the same taxa). *First observation of this virus taxon in the corresponding algal clade. The levi-like sequence that likely infects a bacterial host was excluded.

We found evidence for RNA viruses—that is, hits to the viral RdRp—in eight of the 19 major groups of microalgae, without detectable virus/algal taxon specificity (Fig. 4).

The distribution of RNA viruses was highly heterogeneous among the microalgae studied, with a large representation in the Bacillariophyta (i.e. diatoms), Dinophyceae, and Haptophyceae, with only a few or no viruses in the other taxa (Fig. 4). It is important to note that the number of viruses is strongly associated with the number of libraries analysed and thus likely depicts a limit of detection imposed by small sample sizes in some groups (i.e. large numbers of transcriptomes are available for the Bacillariophyta, Dinophyceae, and Haptophyceae).

### 3.4 Positive-sense RNA viruses (ssRNA+)

Eleven of the 30 viruses discovered here showed clear homology to three of the four families of the phylum *Lenarviricota* of ssRNA+ viruses: the *Leviviridae*, the *Narnaviridae*, and the *Mitoviridae* (Table 1). In all cases, levels of RdRp identities to the closest homologs were <60 per cent, reflecting high levels of sequence divergence and leading us to propose that these eleven sequences are novel viral species (Table 1).

#### 3.4.1 Narnaviridae*-like sequences*

Three RdRp-containing contigs—denoted Amphitrite narna-like virus, Poseidon narna-like virus, and Halia narna-like virus—were related to the *Narnaviridae*, occupying diverse positions in a phylogeny of this family (Figs 2 and 5). While the closest homologs of these narna-like viruses were in fungi, oomycete (protist), and marine arthropod samples, all three samples that contain these viruses are Bacillariophyta species (*Astrosyne radiata* and *Pseudo-nitzschia pungens*) (Table 1, Fig. 5). As their genome sequences share ∼12 per cent pairwise identity with other *Narnaviridae*, we propose that Amphitrite narna-like virus, Poseidon narna-like virus, and Halia narna-like virus represent novel species within the genus *Narnavirus*.

#### 3.4.2 Mitoviridae*-like sequences*

Seven RdRp protein sequences, retrieved from diverse algae host lineages—Rhodophyta, Haptophyta, Chlorophyta, and Bacillariophyta—were related to members of the *Mitoviridae* (Fig. 5). According to their placement in the *Mitoviridae* phylogeny and genetic distances, these seven new viruses are potential members of the genus *Mitovirus* (Fig. 5, Table 1). All have similar genome organizations, with the exception of one with a genome that seemingly encodes a single RdRp-containing ORF (Fig. 5). It is also notable that the RdRp-encoding ORFs from Aiolos mito-like virus, Asopus mito-like virus, and Daimones mito-like virus can only be predicted using the mitochondrial code (Fig. 5).

#### 3.4.3 Leviviridae*-like sequences*

One viral RdRp-like hit, in the Chlorophyta species *Pycnococcus provasolii*, is related to some bacteria-infecting *Leviviridae*, and based on the levels of sequence identity, this likely constitutes a new genus in this family (Table 1). As there were some bacterial reads in the *Pycnococcus provasolii* samples (MMETSP1471) (Fig. 3B), it is likely that this Triton levi-like virus sequence infects bacteria (Actinobacteria or Proteobacteria-like) also present in the culture rather than *Pycnococcus provasolii*.

#### 3.4.4 Picornavirales*-like sequences*

**Figure 6.**
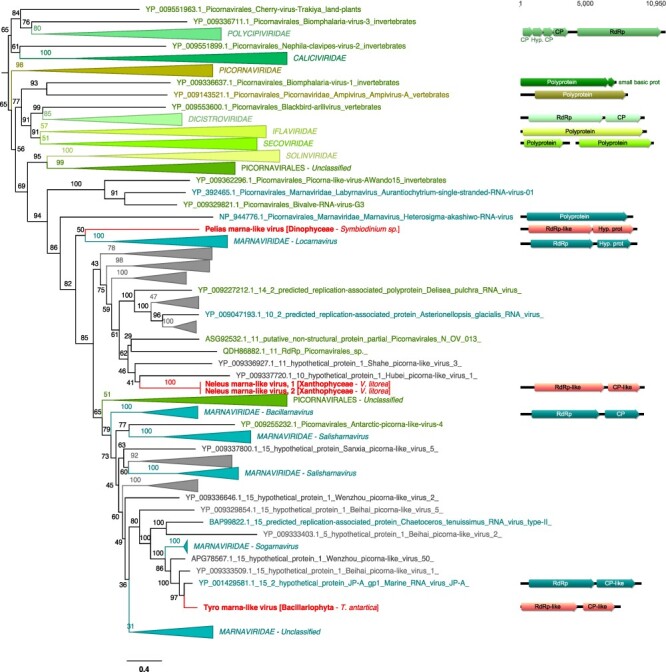
Phylogenetic placement of the newly described RNA virus sequences in the order *Picornavirales*. Left, Maximum likelihood (ML) phylogeny of the *Picornavirus* RdRp (LG + F + R10 amino acid substitution model). Newly described viruses are indicated in red. Algae host taxon and species are specified in brackets. Branch labels = bootstrap support (%). The tree is mid-point rooted for clarity only. Right, genomic organization of newly described viruses (red), closest homologs, and the following *Picornavirales* order RefSeq representatives: Solenopsis invicta virus 2 (NC_039236; *Polycipiviridae*), Porcine enteric sapovirus (NC_000940; *Caliciviridae*), foot-and-mouth disease virus—type O (NC_039210; *Picornaviridae*), acute bee paralysis virus (NC_002548; *Dicistroviridae*), infectious flacherie virus (NC_003781; *Iflaviridae*), and cowpea severe mosaic virus (NC_003544/NC_003545; *Secoviridae*). For clarity, some lineages were collapsed (a non-collapsed version of the tree is available as Supplementary Information).

Three sequences—denoted Pelias marna-like virus, Neleus marna-like virus, and Tyro marna-like virus—were identified in diverse cultures belonging to various taxa (Fig. 4): *Symbiodinium sp.* (Dinophyceae), *Vaucheria litorea* (Xanthophyceae), and *Thalassiothrix antarctica* (Bacillariophyta). These viruses exhibit sequence similarity with ssRNA+ viruses from the order *Picornavirales*, falling within the large algal-associated family *Marnaviridae* (Fig. 2C). Based on their positions in the phylogeny and the level of sequence divergence, Pelias marna-like virus could constitute a new genus in the *Marnaviridae*, while Neleus marna-like virus and Tyro marna-like virus are likely members of the genera *Kusarnavirus* and *Sogarnavirus*, respectively (Fig. 6, Table 1). They also seem to share similar genome lengths and organizations as their closest relatives (Fig. 6).

### 3.5 dsRNA viruses

**Figure 7.**
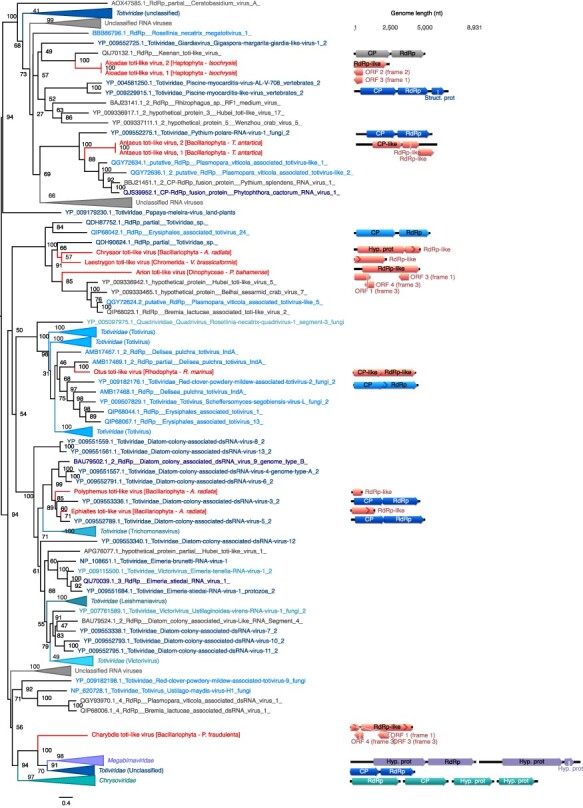
Phylogenetic position of the newly described RNA virus sequences among the *Ghabrivirales*. Left, Maximum likelihood (ML) phylogeny of the *Ghabrivirales* RdRp (LG + F + R10 amino acid substitution model). Newly described viruses are indicated in red. Algae host taxon and species are specified in brackets. Branch labels = bootstrap support (%). The tree is mid-point rooted for clarity only. Right, genomic organization of the newly described viruses (red), closest homologs and the following representative *Ghabrivirales*: Rosellinia necatrix megabirnavirus 1/W779 (NC_013462/NC_013463; *Megabirnaviridae*), Tuber aestivum virus 1 (NC_038698; *Totiviridae*), and Penicillium chrysogenum virus (NC_007539/NC_007540/NC_007541/NC_007542; *Chrysovirida*e). For clarity, some lineages were collapsed (a non-collapsed version of the tree is available as Supplementary Material).

Almost a third of the RNA viruses newly reported here were related to dsRNA viruses of the family *Totiviridae* (Fig. 2D). The single exception was a more divergent Charybdis toti-like virus, the exact placement of which within the order *Ghabrivirales* was unclear as it occupied a basal position in the phylogeny with only limited sequence similarity to related viruses (∼30 per cent at the RdRp protein level) (Fig. 7, Table 1).

Aloadae toti-like virus, found in Haptophyta *Isochrysis sp*, groups with the protist-associated *Giardiavirus* genus (*Totiviridae*) and more surprisingly with Keenan toti-like virus identified in ectoparasitic flies, although with very high levels of sequence divergence (Fig. 7; Table 1). Similarly, Chrysaor toti-like virus, Laestrygon toti-like virus, and Arion toti-like virus, retrieved from Bacillariophyta, Chromerid, and Dinophyceae, respectively, form a clade with *Totiviridae*-like sequences identified in either marine arthropods or oomycete protists (Fig. 7). While these likely constitute a new genus within the *Totiviridae*, their host remains uncertain. Antaeus toti-like virus, retrieved from the Bacillariophyta *T. antarctica*, groups with Pythium polare RNA virus 1 that infects the oomycete *Pythium polare*, confirming the presence of a polar stramenopile clade in the *Totiviridae*. Otus toti-like virus, identified in the Rhodophyta *Rhodosorus marinus*, clusters with the Delisea pulchra totivirus identified in the Rhodophyta (Fig. 7). Two additional toti-like viruses—Polyphemus toti-like virus and Ephialtes toti-like virus—were identified in *A. radiata* (Bacillariophyta) and, together with the diatom colony-associated dsRNA viruses, form a new clade, and likely genus, associated with Bacillariophyta (diatoms) (Fig. 7).

Strong similarities in genome organization were observed between the Otus toti-like virus and Antaeus toti-like virus and their toti-like homologs, with a potential single segment encoding a coat protein (CP) in 5ʹ and a RdRp in 3ʹ (Fig. 7). As Charybdis toti-like virus, Chrysaor toti-like virus, Laestrygon toti-like virus, Arion toti-like virus, Polyphemus toti-like virus, and Ephialtes toti-like virus all had partial genomes, we were unable to determine their genomic organization, aside from the observation that they all fell within the unsegmented *Totiviridae*. Such an assumption cannot be made for Charybdis toti-like virus, because of its basal position within the *Ghabrivirales*.

We identified six RdRp hits to members of the *Durnavirales* order of dsRNA virus (Fig. 2C). With the exception of Aethusa amalga-like virus and Aegean partiti-like virus, their exact phylogenetic position within the six families that comprise this order (*Partitiviridae, Hypoviridae, Picobirnaviridae*, and *Amalgaviridae*) is unclear (Fig. 8). Moreover, these sequences seemingly have no association with specific microalgal groups, being observed in species of Rhodophyta, Bolidophyceae, Bacillariophyta, Chlorophyta, and Dinophyceae (Fig. 4). Aethusa amalga-like virus, retrieved from the Rhodophyta *R. marinus*, is clearly related to the *Amalgaviridae* (Figs 2 and 8) and displays 43 per cent identity in the RdRp to *Zygosaccharomyces bailii virus Z* identified in fungi (Table 1). Whether this constitutes a new genus within the *Amalgaviridae* remains to be determined.

Three other viruses, Benthesicyme durna-like virus, Herophile durna-like virus, and Cymopoleia durna-like virus, were related to the Amalga-like lacheneauvirus and Amalga-like chassivirus, both previously identified in cultures of *Ostreobium* sp. (Chlorophyta), and that fell between the *Amalgaviridae* and *Partitiviridae* families in our phylogenetic analysis (Fig. 8). The genomic sequences for Benthesicyme durna-like virus, Herophile durna-like virus, and Cymopoleia durna-like virus were likely partial such that their organization, particularly whether they comprise one or two segments, could not be established (Fig. 8).

Aegean partiti-like virus falls in the *Partitiviridae*, grouping with the Partiti-like lacotivirus, Partiti-like allasinovirus, Partiti-like Adriusvirus, and Bryopsis cinicola chloroplast dsRNA, all of which are associated with Ulvophyceae algae (Fig. 8). The presence of Aegean partiti-like virus in *Tetraselmis chuii* (Chlorophyta) strongly supports the existence of a Chlorophyta-infecting partiti-like viral genus. Assuming a homologous genome organization, the genome of Aegean partiti-like virus would comprise a single segment encoding a RdRp in its 5ʹ region as well as a hypothetical protein, potentially a CP, in the 3ʹ region. Whether Aegean partiti-like virus is associated with the host chloroplast remains uncertain. Finally, Ourea durna-like virus is highly divergent and falls basal to the bi-segmented *Partitiviridae* (Fig. 8). However, considering the length and the single ORF organization of the partial genomic sequence retrieved, it is likely that a second segment encoding a CP may not have been detected due to extensive sequence divergence.

### 3.6 **Negative-sense viruses (ssRNA** **−**)

**Figure 9.**
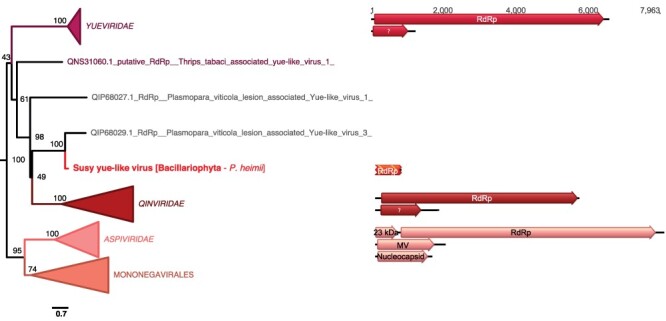
Position of the newly described RNA virus in the phylum *Haploviricotina.* Left, Maximum likelihood (ML) phylogeny of the *Haploviricotinia* RdRp (LG + F + R10 amino acid substitution model). The virus newly described here is shown in red. Algae host taxon and species are specified in brackets. Branch labels = bootstrap support (%). The tree is mid-point rooted for clarity only. Right, genomic organization of the newly described virus (red) and the following homologs representatives: Shahe yuevirus-like virus 1 (NC_033289/NC_033290; *Yueviridae*), Beihai sesarmid crab virus 4 (NC_032274/NC_032272; *Qinviridae*), and Blueberry mosaic-associated virus (NC_033754/NC_036634/NC_036635; *Aspiviridae*). For clarity, some lineages were collapsed (a non-collapsed version of the tree is available as Supplementary Information).

A novel RdRp sequence, Susy yue-like virus, was identified in the *Pseudo-nitzschia heimii* (Bacillariophyta) culture, although at a very low read abundance (five reads/million) such that any host assignment can only be made with caution. This putative virus clusters among the ssRNA− *Haploviricotina*, falling between the *Qinviridae* and the *Yueviridae* families (Fig. 9). Considering the length of the RdRp segment and the bi-segmented genome organization of the *Qinviridae* and *Yueviridae* (Fig. 9), it is likely that the Susy yue-like virus genome is partial. In a similar manner to the *Qinviridae*, Susy yue-like virus has an IDD (Ile-Asp-Asp) sequence motif instead of the common GDD (Gly-Asp-Asp) triad in the catalytic core of its RNA virus replicase (RdRp), although any functional implications are unclear.

### 3.7 Detection of divergent RNA viruses based on RdRp motifs and structural features

To identify RNA viruses at lower levels of homology than obtained using BLAST-based methods, we conducted an extensive analysis utilizing RdRp protein functional motifs and structural features on all the BLAST-unannotated sequences: this accounted for 10–34 per cent of the total predicted ORFs of at least 200 amino acid residues in length (Fig. S2).

A very large proportion of sequences retained from our combined RdRp-based HMM analysis were false-positive hits as they were either detected as eukaryotic-like sequences using Phyre2 or were too distant to be safely considered as an RdRp (i.e. unreliable alignment and no detection of RdRp catalytic motifs) (Table S3). However, five RdRp-like candidates were identified following manual curation. While no robust RdRp-like signal could be detected using Phyre2 (i.e. prediction confidence scores below 90 per cent) (Table S3), the presence of a significant HMM-detected homology with the PROSITE PS50507 profile (i.e. RdRp of ssRNA+ virus catalytic domain profile; Table S2) enabled us to further analyse these candidates as potential viral RdRps.

Four of these RdRps came from the genus *Bigelowiella*, and three (MMETSP0045_DN12861, MMETSP1054_DN18666, and MMETSP1052_DN19445) shared high identity levels (>90 per cent at both protein and nucleotide levels; Table 2). Although the PROSITE PS50507 profiles were built from ssRNA+ RdRp sequences, the IDD C-motif exhibited by these four RdRp-like candidates is found in the ssRNA− *Qinviridae*-like viruses as well as the new Susy yue-like virus (MMETSP1423). Importantly, however, the nucleotide sequences of these RdRp-encoding candidates exhibited a strong match (e-value <1e-90), with a genome contig (BIGNAscaffold_41_Cont1731) from the *Bigelowiella natans* genome (GCA_000320545.1). Hence, rather than representing an exogenous RNA virus, the RdRp hit in these cases most likely constitutes an EVE indicative of a past, and likely ancient, infection event.

**Table 2.** RdRp-like hits retrieved from the HMM-profile and Phyre2 analyses. Presence of the A, B, and C motifs is noted along with the sequence of the C-motif.

Contig ID	Taxon	RdRp profile	e-value	A	B	C	Phyre2 confid%	%ID	Hit info
MMETSP1359_DN14104 _c0_g1_i1_len843_1	*Bigelowiella longifila* (Cercozoa)	PS50507	6.0e-07	Yes	?	IDD	64.2	16	PDB header:t ransferase
MMETSP0045_DN12861 _c0_g1_i1_len664_1	*Bigelowiella natans* (Cercozoa)	PS50507	8.7e-06	Yes	?	IDD	40.7	24	DNA/RNA polymerases
MMETSP1054_DN18666 _c0_g1_i1_len657_1	*Bigelowiella natans* (Cercozoa)	PS50507	8.9e-06	Yes	?	IDD	41.6	24	DNA/RNA polymerases
MMETSP1052_DN19445 _c0_g1_i1_len738_1	*Bigelowiella natans* (Cercozoa)	PS50507	1.0e-05	Yes	?	IDD	40.4	24	DNA/RNA polymerases
MMETSP0202_DN4292 _c0_g1_i1_len814_1	*Karenia brevis* (Dinophyceae)	PS50507	4.6e-05	Yes	?	GDT	56.7	17	PDB header: hydrolase

Finally, in the case of the distant RdRp-like signal in MMETSP0202_DN4292, no GDT sequence at motif C could be identified in an expansive RdRp data set (Wolf et al. 2018). Hence, it is unclear if MMETSP0202_DN4292 represents a true viral RdRp or a false-positive hit.

## 4. Discussion

With the discovery of thirty new and divergent viruses, twenty-nine of which are likely to infect algae species in which no viruses have previously been reported, this study greatly extends our knowledge of the microalgae RNA virosphere and demonstrates the potential of protists to be major reservoirs for novel RNA viruses.

Despite the viral diversity documented, it is striking that only 6 per cent (33 of 570) of the transcriptomes analysed here contained evidence of an RNA virus, far lower than equivalent studies of single organisms (Shi et al. 2016, 2018; Geoghegan et al. 2018). The use of clonal and purified cultures is expected to greatly reduce the number of viruses compared to direct environmental samples, by limiting the number of host cells investigated and preventing the sequencing of co-circulating viruses as well as those infecting other microorganisms in the environment. However, this relative paucity of RNA viruses could also reflect methodological limitations. The lack of rRNA depletion in library processing leads to a reduction in the number of non-rRNA transcripts, including those from viruses. Indeed, most of the viruses reported here display very low transcript abundance, suggesting that additional RNA viruses may go undetected due to poor sequencing coverage. The limited number of viruses identified likely reflects the high levels of sequence divergence expected for protist viruses compared to those currently available in sequence databases. Indeed, this study has been conducted at the boundary of the detectable virosphere, with many of the viruses identified sharing less than 30–40 per cent sequence identity.

### 4.1 RNA viruses are widespread among lineages of unicellular algae

Our knowledge of RNA viruses associated with microalgae is scarce. The small number reported so far are mostly associated with a subset of algal species from the Bacillariophyta and Chlorophyta, ignoring the wide diversity of microalgae (Fig. 1). We extend this diversity by revealing, for the first time, RNA viruses (i.e. RdRp sequences) in the Haptophyta, Chromeraceae (Alveolates), as well as in the Stramenopiles Xanthophyceae and Bolidophyceae. We also identified new virus-algae clade associations. For example, we present the first observation of *Picornavirales, Ghabrivirales* (*Totiviridae*), and *Durnavirales* (*Partititivridae*) in Dinophyceae cultures, *Lenarviricota* and *Durnavirales* in Rhodophyta cultures, and *Durnavirales* in Bacillariophyta cultures. Importantly, our study also constitutes the first possible observation of a *Muvirales*-like ssRNA− virus in a Bacillariophyta sample, although this requires additional confirmation.

With the exception of *Symbiodinium* sp. for which a ssRNA+ virus was previously reported (Correa, Welsh, and Vega Thurber 2013; Levin et al. 2017), all the viruses described in this study represent the first observation of an RNA virus in each respective host species. In addition, none of the seventy-three microalgal viruses reported previously were identified here. If verified, the first observation of an ssRNA− virus in a Bacillariophyta, together with the previous observation of a bunya-like virus reported in the distantly related Chloroarachniophyte *C. reptans* and bunya-like siRNAs in brown algae (Phaeophyta) (Waldron, Stone, and Obbard 2018), would demonstrate that microalgae can be infected with negative-sense RNA viruses. Interestingly, the related *Qinviridae* and *Yueviridae* have been exclusively identified from metagenomic studies conducted on marine arthropods holobionts, such that algae could constitute the true hosts for most of these viruses (Käfer et al. 2019; Wu et al. 2020). Undoubtedly, the presence of ssRNA− viruses in microbial eukaryotes needs to be further characterized.

### 4.2 *Narnaviridae*-like and *Mitoviridae*-like viruses are common in microalgal cultures

Many of the viruses reported here were from the order *Lenarviricota* that includes the *Narnaviridae* and *Mitoviridae* and often characterized by a single RdRp ORF (Hillman and Cai 2013). Although they were initially thought to be restricted to fungi, these seemingly simple RNA viruses appear to be more widespread than initially thought. *Narnaviridae*-like viruses have recently been associated with a wide range of protist organisms, including protozoan parasites like *Plasmodium vivax* (Akopyants et al. 2016; Lye et al. 2016; Grybchuk et al. 2018; Charon et al. 2019) and the oomycete *Phytophthora infestans* (Cai et al. 2012), while narna-like viruses have been detected in diatoms (Urayama, Takaki, and Nunoura 2016). Similarly, the *Mitoviridae* were considered as exclusively infecting fungi, until the recent discovery of the Chenopodium quinoa mitovirus 1 in a plant and mito-like viruses in the Chlorophyta *Osteobium* sp. (Nerva et al. 2019). The three new narna-like viruses in Bacillariophyta discovered here, as well as the proposal of seven new mitovirus-like species in algal lineages as diverse as Haptophyta, Bacillariophyta, Rhodophyta, and Chlorophyta, provide further evidence for the ubiquity of these viruses in protists.

Whether all the mitoviruses documented here are associated with the mitochondria, as is typical of the *Mitoviridae*, remains to be determined. In addition, while the unique RdRp-encoding segment has already been demonstrated as sufficient for virus infectivity, recent studies have suggested the presence of an additional segment, without an assigned function, in both *Leptomonas seymouri* and *Plasmodium vivax* (Lye et al. 2016; Charon et al. 2019). Whether the viruses newly described here have unsegmented or bipartite genomes is uncertain. Most of the *Lenarviricota*-like sequences described here display ambigrammatic ORFs, with their reverse strand encoding additional ORFs. This feature has already been reported in narnaviruses and could represent a potential solution to extreme genome compaction (Belshaw, Pybus, and Rambaut 2007; DeRisi et al. 2019; Dinan et al. 2020).

The ubiquity of *Mitoviridae* and *Narnaviridae* in eukaryotes is compatible with the protoeukaryotic origins of these viruses and the bacterial *Leviviridae*, such that they are relics of a past endosymbiont infection of a eukaryotic ancestor. According to this scenario, cytoplasmic *Narnaviridae* would have escaped from mitochondria to the more RNA hospitable cytosol (Dolja and Koonin 2018). In addition, *Narnaviridae* and *Mitoviridae* are not associated with cellular membranes (Solórzano et al. 2000), which might reflect their ancient origin from a protoeukaryote ancestor without cellular compartments.

### 4.3 The extension of the *Marnaviridae* to new algal taxa

Most of the algal RNA viruses described to date belong to the order *Picornavirales* (Short et al. 2020), including the *Marnaviridae* that are strongly associated with marine samples or algae cultures (Vlok, Lang, and Suttle 2019). Indeed, the three picorna-like viruses newly identified in this study fell within the *Marnaviridae*. Despite similar genome organizations, these three viruses have relatively high levels of divergence from known *Marnaviridae*, in turn suggesting that the *Marnaviridae* diversity has only been sparsely sampled. While the detection of Neleus marna-like virus and Tyro marna-like virus in Bacillariophyta and Xanthophyceae could reflect the specificity of *Sogarnavirus* and *Kusarnavirus* to Stramenopile algae, the first detection of a *Marnaviridae*-like virus in the Dinophyceae species *Symbiodinium* sp. suggests that the host range of this algal-infecting viral family is not restricted to Stramenopile eukaryotes.

### 4.4 The ancestry of the *Durnavirale*s and *Ghabrivirales* dsRNA viruses

Approximately half of the RNA viruses identified in this study were related to the *Totiviridae* (*Ghabrivirales*) and *Partitiviridae* (*Durnavirales*) families of dsRNA virus. The *Totiviridae* currently comprises 28 formally assigned species in five genera (Lefkowitz et al. 2018; Walker et al. 2020). Interestingly, *Totiviridae* are exclusively associated with unicellular eukaryotes, with two of the five *Totiviridae* genera associated with latent fungal infections (*Totivirus* and *Victorivirus*), while *Trichomonasvirus*, *Giardiavirus*, and *Leishmaniavirus* have been associated with protozoan parasite infections (Lefkowitz et al. 2018).

Each of the *Totiviridae*-like sequences identified here were retrieved from a range of algal hosts spread among diverse branches of the microbial eukaryote tree (Bacillariophyta, Dinophyceae, Haptophyceae, Rhodophyta, and Chromeraceae). In addition, some of the novel viruses identified clusters with totiviruses previously reported in Bacillariophyta diatoms (Sasai et al. 2018; Chiba et al. 2020) and the Rhodophyta *Delisea pulchra* (Lachnit, Thomas, and Steinberg 2016). These observations support the existence of a Bacillariophyta and a Rhodophyta-infecting clade in the genus *Totivirus* that will need to be confirmed with studies of additional species. It was also notable that other toti-like viruses identified here group with viruses found in non-algal hosts, such as invertebrates (ticks, crustaceans), fungi, and protozoan parasites. While host mis-annotations cannot be formally excluded, the presence of *Totiviridae* in protozoan parasites, fungi, and algae could signify that the host range of the *Totiviridae* is far larger than appreciated.

Six dsRNA-like new viruses identified here show clear homology with those of the order *Durnavirales*, including the *Partitiviridae* and the *Amalgaviridae* that comprise bi-segmented and unsegmented dsRNA viruses, respectively. The *Partitiviridae* are classified into five genera mainly associated with plants and fungi, although more recently with oomycetes (Shiba et al. 2018) and to Apicomplexa (Nibert et al. 2009). The *Amalgaviridae* comprise two genera associated with either fungi (*Zybavirus* genus) or land plants (*Amalgavirus* genus) (Park et al. 2018; Walker et al. 2020). In addition to the recent association of newly described partiti- and amalgavirus-like viruses in the microalgae *Ostreobium* sp. (Cholorophyta) (Charon et al. 2020), our identification of these novel and divergent *Durnavirales*-like viruses in several distant algae taxa again suggests that host range for this viral order has been underestimated.

### 4.5 Limitations to virus discovery and inferring virus–host relationships

A key element of this study was the use of mono-strain cultures, which were axenic whenever possible, enabling more accurate virus–host assignments. While Bacteria, and to a lesser extent, Archaea, were present in the non-axenic cultures, the placement of most of the newly described viruses within eukaryotic-infecting viral families clearly supports their association with algae. Despite this, some of the newly described viruses were associated with viral lineages traditionally associated with fungal or metazoan hosts. This likely reflects the lack of representation of microalgal viruses in current sequence databases or a mis-annotation to secondary metazoan host, particularly given the recent efforts to describe the fungal virome (Xie and Jiang 2014; Ghabrial et al. 2015; Marzano et al. 2016; Deakin et al. 2017). Similarly, many of the newly identified viruses share homology with viruses identified in metagenomics studies of marine invertebrates (Shi et al. 2016). Such similarities to holobiont virome studies should be treated with caution, as the viruses reported could be infecting symbionts, eukaryotic parasites, or bacteria that are also present in these samples (Dolja and Koonin 2018). Marine invertebrate organisms are also important ocean filters and virus removers (Welsh et al. 2020), again compatible with the idea that some of the viruses identified here may infect other marine organisms.

We also attempted to identify more distant RNA viruses using a protein profile and structural-based approach. However, no remote RNA virus signals could be confidently detected, although a distant EVE in *Bigelowiella* was identified. While the de novo prediction of protein three-dimensional structures has undergone major improvements in the last decade (Callaway 2020), detecting robust homology strongly relies on comparisons with already known protein structures (Kelley et al. 2015). Critically, however, only a very limited number of non-human viruses are available among the viral proteins deposited in the Protein Data Bank, representing a major roadblock in detecting highly divergent RdRps. Indeed, a better characterization of RdRp structures combined with the enrichment of RdRp motif and profile databases will help counter the challenge posed by the high levels of sequence divergence in protist samples and the concomitant loss of detectable evolutionary signal. In addition, the high percentage of false positives in the HMM analysis highlights the need to increase and optimize the sensitivity and stringency of such methods.

Finally, while our study greatly extends our knowledge of RNA virus diversity among unicellular eukaryotes, experimental confirmation is needed to formally assign viruses to their specific microalgae hosts and to assess the impact of viral infection on host biology.

## Supplementary Material

veab070_Supp

## Data Availability

The raw transcriptome data used in this study are available on the NCBI Sequence Read Archive (SRA) at BioProject PRJNA231566 (individual accessions are provided in Table S1). The consensus nucleotide and amino acid sequences (fasta format) for the viruses identified in this study, multiple sequence alignments, and resultant phylogenetic trees are available at https://github.com/JustineCharon/MMETSP_RNA_virus_data.
