# Supplementary figures and images for: Revealing RNA virus diversity and evolution in unicellular algae transcriptomes
Source: Virus Evol. 2021 Aug 14;7(2):veab070. doi: 10.1093/ve/veab070 (PMC9927876; doi:10.1093/ve/veab070)

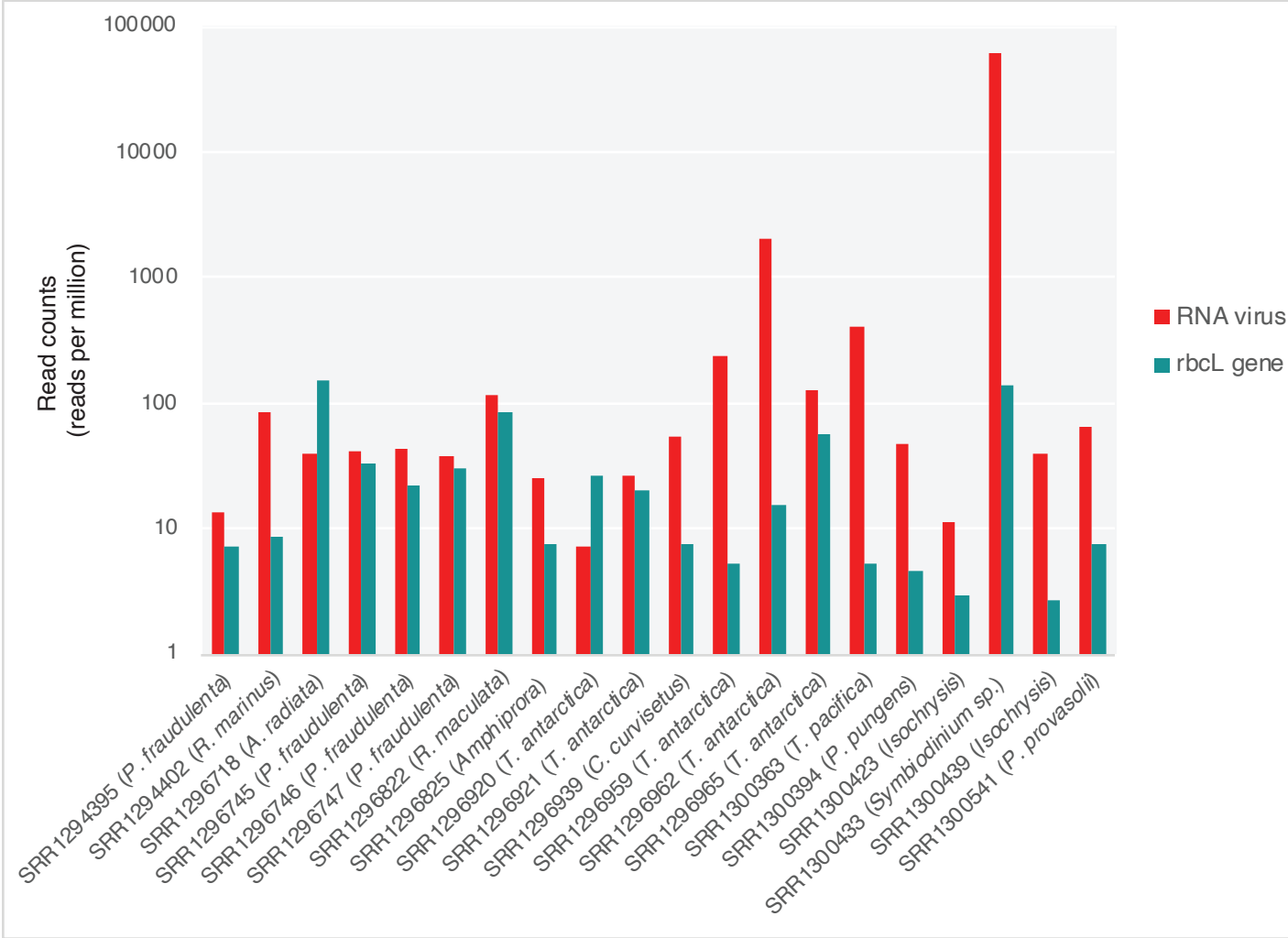

Supplement: veab070_Supp [file veab070_supp.zip › Charon.Figure S1.pdf]

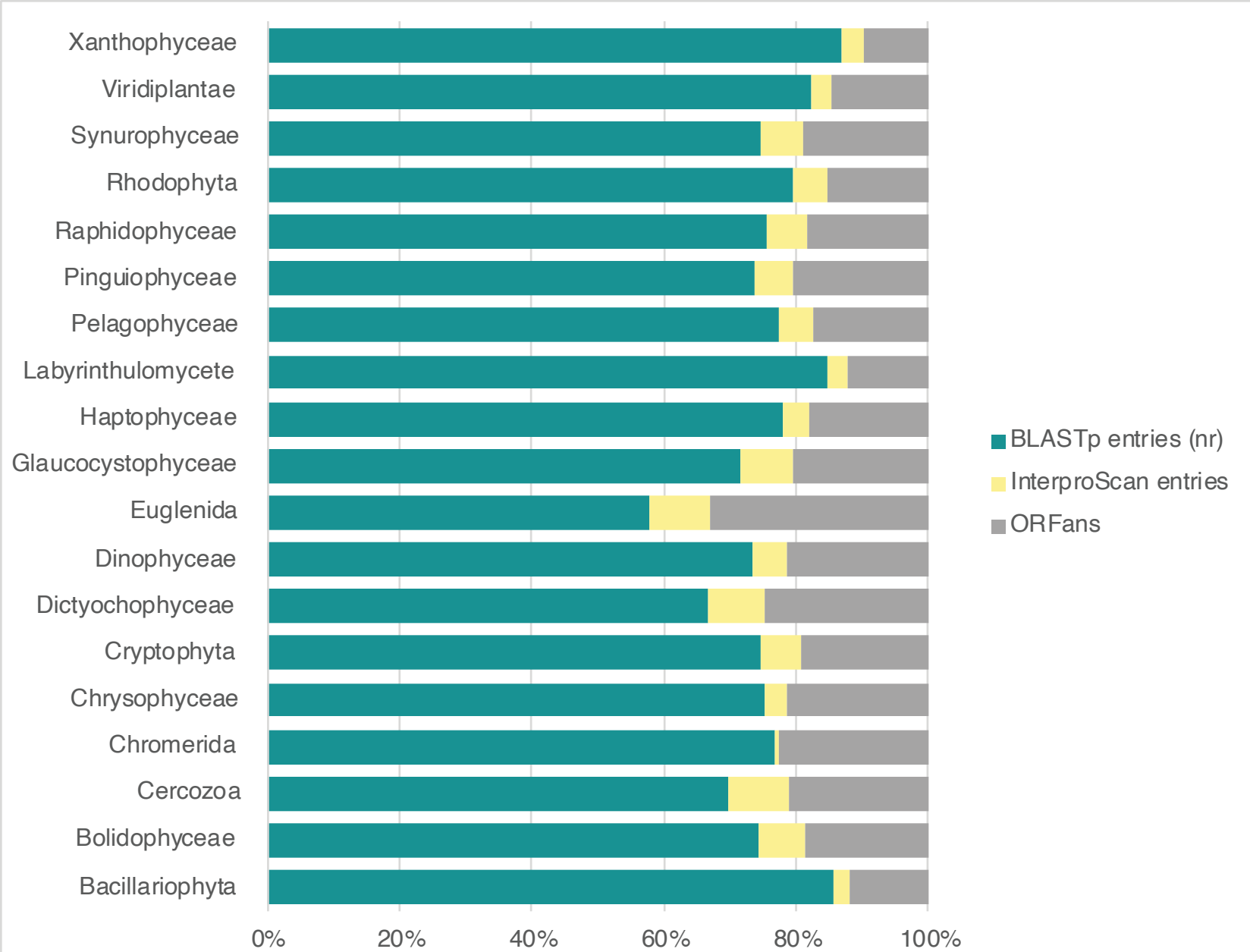

Supplement: veab070_Supp [file veab070_supp.zip › Charon.Figure S2.pdf]
